# LEISURE ACTIVITY AND POSITIVE MOOD ACROSS ADULTHOOD: RESULTS FROM THE KOREAN TIME USE SURVEY

**DOI:** 10.1093/geroni/igae098.3485

**Published:** 2024-12-31

**Authors:** Seung-Eun Cha, Kyungmin Kim, Hyejoong Kim

**Affiliations:** University of Suwon, Hwasung si, Kyonggi-do, Republic of Korea; Seoul National University, Seoul, Republic of Korea; Gachon University, Seongnam-si, Kyonggi-do, Republic of Korea

## Abstract

Adults in middle and later life may look forward to dedicating more time to outdoor leisure activities, which can contribute to their health and well-being. This study utilized 2-day time diaries from the 2019 Korean Time Use Survey (respondent N = 22,821; age 20−84) to examine how Korean adults engage in various outdoor activities (e.g., social visits, attending cultural events, and exercising) and whether their activity engagement (indicated by outdoor activity diversity and total hours of outdoor activity) is associated with positive mood. Further, we investigated whether these associations varied by age groups (age 20–39, 40–64, and 65–84), considering differences in family and work demands. We accounted for respondents’ total TV viewing hours, total “doing-nothing” hours, and sociodemographic characteristics as control variables in the models for positive mood. The findings showed that total hours of outdoor activities and watching TV were positively associated with a positive mood in younger adults, supporting the psychological benefits of detachment from work. Yet, for older individuals, diversity in outdoor activities was positively associated with positive mood, while total hours of outdoor activities were not significant—which indicates that engaging in various activities throughout the day can be beneficial for psychological well-being in later life. Notably, we found that total TV viewing hours were negatively associated with the positive mood of older adults. This study sheds light on the differential impacts of leisure activities based on life stages, underscoring the importance of diverse leisure activities for the psychological health of older individuals.

